# How do contract performance rates affect entrepreneurs’ risk-averse attitudes? Evidence from China

**DOI:** 10.3389/fpsyg.2023.1112344

**Published:** 2023-03-10

**Authors:** Zenan Sun, Shen Lu, Man Huang, Jincai Zhuang, Andrea Maria Vaca Lucero, Charles Dwumfour Osei

**Affiliations:** School of Management, Jiangsu University, Zhenjiang, China

**Keywords:** contract enforcement efficiency, entrepreneurial, risk attitudes, risk aversion, business growth, moderators, mediators

## Abstract

**Introduction:**

Entrepreneurs’ attitudes toward risk is one of the most critical factors influencing business growth and economic development. Therefore, addressing the influencing factors and formation mechanisms of entrepreneurs’ risk attitudes has become a crucial research endeavor. In this paper, we examine how contract performance rates affect entrepreneurs’ risk attitudes through the mediating effect of subjective well-being as well as assess the moderating effect of the regional business environment on this relationship.

**Methods:**

The ordered probit regression technique was employed to analyze the data obtained from 3,660 sampled respondents from the 2019 China Household Finance Survey. All analysis was performed using Stata 15.0.

**Results:**

The empirical results show that contract performance rates have a substantial positive indirect effect on entrepreneurs’ degree of risk aversion through improved subjective well-being. The regional business environment plays a negative regulatory role in the relationship between contract performance rates and entrepreneurs’ risk aversion. Furthermore, urban–rural heterogeneity appears to consistently determine the extent of the influence of contract performance rates on entrepreneurs’ risk attitudes.

**Conclusion:**

To reduce entrepreneurs’ risk aversion and enhance social and economic activity, the government should improve regional business environments by taking specific measures. Our study contributes to the empirical understanding of entrepreneurs’ investment decisions in the context of urban and rural environments.

## 1. Introduction

To date, it has been indicated that individual risk attitudes are linked to various types of behaviors, including saving, consumption, investment, and career choices (Djankov et al., 2006; Caliendo et al., 2010; Pfeifer, 2011). More importantly, as a critical factor, individual risk attitudes are expected to influence business development as entrepreneurs’ risk attitudes, through their strategic choices, affect the behaviors of their firms (Soreide, 2009; Dvorsky et al., 2022). For example, in practice, entrepreneurs’ risk aversion often leads them to become conservative (Taofeeq et al., 2022). Furthermore, risk aversion causes entrepreneurs to refuse to accept new technologies and develop new products or markets (Kashan et al., 2021; Bai and Jia, 2022). As a result, entrepreneurs’ risk attitudes may indirectly account for regional economic growth and social development at the macro level (Pflueger et al., 2018). Therefore, addressing the influencing factors and formation mechanisms of entrepreneurs’ risk attitudes has become a crucial research endeavor.

Regarding the current research findings, most studies on the factors influencing entrepreneurs’ risk attitudes have focused on individual traits. Numerous studies have pointed out that gender, age, and cognitive ability influence entrepreneurs’ risk attitudes (see, e.g., Yordanova and Alexandrova-Boshnakova, 2011; Sepulveda and Bonilla, 2014; Block et al., 2015; Liu et al., 2021). It is worth noting that running a business is entrepreneurs’ primary means of earning income. Hence, their business situation often affects their mood and social status and potentially further influences their risk attitudes. For example, a recent study of small retail businesses in Vietnam noted that entrepreneurs facing a financially difficult situation in their business showed less risk aversion than those facing fewer financial shocks in the placebo group (Dalton et al., 2020). However, the business situations faced by many firms are often more complex in practice. Therefore, the factors that influence entrepreneurs’ risk attitudes at the business level are more diverse. Recent scholars have shown that transaction costs significantly affect the situation of many businesses (e.g., Andreassen et al., 2018). In turn, contract performance rates are one of the most realistic expressions of business transaction costs (Williamson, 1979). Hence, the present study specifically explores the relationship between contract performance rates and entrepreneurs’ risk attitudes as well as its influencing mechanisms.

This paper employs the data from the 2019 China Household Finance Survey (Gan et al., 2014). Initially, the ordered probit (oprobit) model is used for the benchmark regression analysis. Then, this paper explores its mechanism by constructing intermediary and regulatory effects models. Finally, the robustness of the regression model is tested using the substitution estimation method, which further verifies the reliability of the conclusions of this paper.

Compared with previous studies, this paper makes the following contributions: (1) we demonstrate the effect of improving contract performance rates on entrepreneurs’ risk attitudes, which expands the research on individual risk attitudes and business management and (2) by constructing intermediary and regulatory effect models, this study further tests the mediating effect of subjective well-being and the moderating effect of the regional business environment. These findings expand the relationship between contract performance rates and entrepreneurs’ risk attitudes. Meanwhile, it also provides a theoretical basis for formulating relevant policies.

The remaining sections of this paper are organized as follows: Section 2 discusses the relevant literature and develops the hypotheses; the data construction, sampling, empirical specification, and estimation procedures are discussed in Sections 3, 4 presents the main empirical results and discussion, while Section 5 outlines the robustness checks; the results of the heterogeneity analysis are presented and discussed in Section 6; and finally, the conclusions and policy recommendations are presented in Section 7.

## 2. Literature review and research hypotheses

### 2.1. Contract performance rate

Performance is one of the stages of the contract life cycle (Guth et al., 2003). The “execution” of the contract takes place in this stage—that is, the contract parties exercise their rights and fulfill their duties under the corresponding contract. It is worth noting that although the time nodes and the chronological sequence of all contract parties’ actions are generally specified in the contract, in practice, contract parties always delay the performance of their duties for various reasons, especially in traditional industries (Duary et al., 2022; Kovach et al., 2023). For this reason, we propose a “contract performance rate” to describe the speed at which a contracting party fulfills their contractual duties.

Under certain external conditions, shorter inter-firm lead times for product delivery and payment imply lower additional costs to conclude transactions. Hence, the rate of inter-firm contract performance can be considered a realistic representation of their transaction costs (Williamson, 1979). Previous research on contract performance rates has explored their relationship with business performance and firm behavior, such as the return on assets (Manullang et al., 2020; Hasanudin et al., 2022) and profitability (Pratama et al., 2021) of firms as well as the willingness of private suppliers to engage in public-private partnership projects (Hartman et al., 2020). While existing studies have examined the effect of contract fulfillment on firm behavior, few studies have combined contract performance rates with entrepreneurs’ risk attitudes.

### 2.2. Risk attitude

Risk attitude is the amount of risk one will endure for a potential benefit, and it is typically described as either risk-seeking or risk-averse based on decisions related to gambling parameters such as potential gain probability, gain amount, loss amount, loss probability, variance, entropy, framing effects, and expected value (Purcell et al., 2022). Although classical economic theory generally assumes that individual risk attitudes are exogenously determined such that individual risk attitudes should always remain constant regardless of time, this assumption has been challenged in recent years. Related studies point out that personal risk attitudes may change under the influence of certain factors (e.g., Schildberg-Hoerisch, 2018).

One stream of the existing literature focuses on the influence of individuals’ inherent characteristics on their risk attitudes. Most findings from such studies have concluded that individual risk attitudes are related to personality (Bucciol and Zarri, 2017), cognitive ability (Bonsang and Dohmen, 2015), education (Kapteyn and Teppa, 2011), and other factors (Zhang et al., 2021b). Other scholars have considered the influence of individuals’ past experiences, context, and current state of life on their risk attitudes. Related studies point out marital status (Arrondel and Lefebvre, 2001; Browne et al., 2022; Xie et al., 2022), health status (Hammitt et al., 2009; Ruggeri and Drago, 2021), parental status (Görlitz and Tamm, 2020), income uncertainty (Guiso and Paiella, 2008), work situation (Sahm, 2012; Shlomit et al., 2012; Hetschko and Preuss, 2020), and natural disasters (Reynaud and Aubert, 2022) as the main influencing factors of individual risk attitudes.

### 2.3. Contract performance rates and entrepreneurs’ risk attitudes

As stated above, the rate of inter-firm contract performance can be considered a realistic representation of transaction costs (Williamson, 1979). Furthermore, a low contract performance rate makes it necessary for firms to incur additional costs to obtain the capital needed to operate the business from outside sources, which can affect its profitability (Tang et al., 2007). In extreme cases, companies may become overly indebted and even go bankrupt. Running a business is entrepreneurs’ primary source of income. Therefore, a low contract performance rate will affect entrepreneurs’ income and thus standard of living. Meanwhile, past studies have suggested that individuals’ risk attitudes may change in response to changes in income, standard of living, and social status (e.g., Guiso and Paiella, 2008). Thus, this research concludes that the contract performance rate in entrepreneurs’ industry will directly affect their risk attitudes.

In addition, prospect theory, which was proposed first by Kahneman and Tversky (1979), argues that individuals underweight probable outcomes in comparison with outcomes that are certain. They refer to this phenomenon as the certainty effect and point out that it brings about risk aversion in choices involving certain gains and risk-seeking in choices involving certain losses (Kahneman and Tversky, 1979). In other words, people in a positive affective state can actually be more risk-averse—especially in the presence of high stakes—while they are more risk-seeking if in a negative affective state. In practice, when firms are in a situation in which contract performance rates are high, they are generally able to operate in a stable manner. In such cases, entrepreneurs will perceive themselves as relative winners, and risk-taking may change their current state; thus, they may then exhibit risk-averse characteristics. Conversely, when firms are in a situation in which contract performance rates are low, the survival and growth of the firm is more uncertain. Entrepreneurs facing this condition will perceive themselves as being in a state of relative loss and thus risk-taking may improve their current situation; thus, they may then exhibit risk-seeking characteristics. As a result, in this paper we hypothesize that:

Hypothesis 1: Increased contract performance rates directly reinforce entrepreneurs’ risk-averse attitudes.

### 2.4. The mediating role of subjective well-being

The concept of subjective well-being (SWB) refers to people’s emotional and cognitive evaluation of their own lives and is interchangeable with the notions of happiness, utility, life satisfaction, and welfare (Diener et al., 2003; Easterlin, 2003). Related studies point out that individuals’ subjective well-being is related to their sense of security and life certainty. For instance, Chirumbolo et al. noted negative relationships between job insecurity, life uncertainty, and individual well-being (Chirumbolo et al., 2022). Moreover, Howell et al. found that uncertainty about one’s COVID−19 risk predicted more significant worry about the virus and one’s risk of contracting it and that greater worry would, in turn, predict poorer well-being (Howell et al., 2022).

For entrepreneurs, a low contract performance rate will not only directly affect their income but also expose their businesses to more significant uncertainty (Tang et al., 2007; Manullang et al., 2020). Based on this, we suggest that contract performance efficiency is positively related to entrepreneurs’ subjective well-being.

Furthermore, a higher level of well-being generally implies that people are satisfied with the status quo. Similarly, based on prospect theory, people who are satisfied with the status quo will generally not be willing to take risks to change it. On the contrary, when people are highly dissatisfied with the status quo, they are usually more willing to take risks to change it. At the same time, some empirical studies show that people’s risk attitudes to be significantly associated with their happiness (e.g., Phulkerd et al., 2021). Based on this, subjective well-being is positively related to entrepreneurs’ risk attitudes. Hence, in this paper, we hypothesize that:

Hypothesis 2: Increased contract performance rates can strengthen entrepreneurs’ risk-averse attitudes through the mediating effect of subjective well-being.

### 2.5. The regulatory role of the regional business environment

The regional business environment affects the survival and development of enterprises and determines the investment and business activity in a region (Kolasiński, 2015). This paper seeks to explore the moderating effect of the regional business environment in the relationship between contract performance rates and entrepreneurs’ risk attitudes. Related studies point out that optimizing the regional business environment helps to strengthen the local economy and reduce transaction costs (e.g., Wang et al., 2022). In turn, low transaction costs can help stimulate investment and thus promote development and progress. For instance, Shi et al. (2020) find that improved urban informatization helps to reduce transaction costs, which, in turn, plays a positive role in enhancing enterprises’ total productivity factor. Similarly, Yu and Wei (2022) point out that improving the host country’s business environment helps to reduce transaction costs, which consequently enhances their preference for the host country in overseas M&A. Therefore, costs are relatively lower in areas with a better business environment. In turn, the reduced financial burden gives companies the ability to take additional risks.

On the other hand, the regional business environment reflects social trust to a certain extent (Grayson et al., 2008). Put differently, in regions with a favorable business environment, companies are likely to trust their partners more. Therefore, entrepreneurs in better business environments are more likely to have confidence in their suppliers and customers despite reduced contract performance rates and are thus relatively more willing to continue to take risks. In summary, this paper proposes that:

Hypothesis 3: The regional business environment plays a regulatory role in the relationship between contract performance rates and entrepreneurs’ risk-averse attitudes.

**Figure 1:**
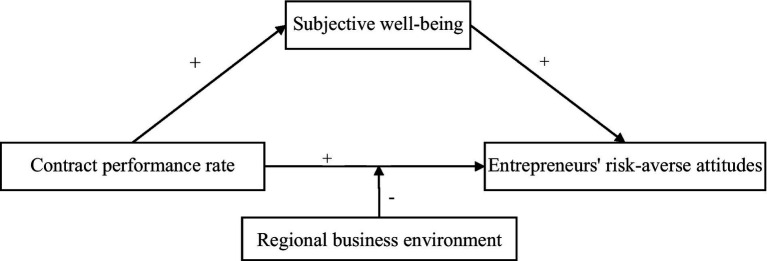
Conceptual model.

Figure 1 presents the hypothesized relationships bet ween the key variables discussed in this paper. The conceptual framework shows both a direct and indirect influence between contract performance rates and entrepreneurs’ risk-averse attitudes through the mediating role of subjective well-being and the moderating role of the regional business environment.

## 3. Data and methodology

### 3.1. Data

#### 3.1.1. Data sources

The empirical data used in this study were obtained from the 2019 China Household Finance Survey (CHFS, 2019). The CHFS database is an interdisciplinary large-scale follow-up survey conducted by the Southwestern University of Finance & Economics to collect relevant information about housing, financial assets, debts, income, insurance, employment, demographic features, etc. The CHFS employs a multistage, random cluster process to draw a sample of about 40,000 households in 29 provinces (excluding Hong Kong, Macao, Taiwan, Tibet, and Hainan) across China.

This paper studies the impact of contract performance rates on entrepreneurs’ risk attitudes. In doing so, the sample data were treated as follows: (1) all employees were excluded from the sampled respondents while retaining only the sampled employers whose occupations are in the business and industrial sectors; (2) the age of the respondents ranged from 18 to 65 years; and (3) samples with severe data loss were also removed. Finally, 3,660 validated samples from the dataset used in this research. It should be noted that STATA software version 15 was used to perform all statistical analyses.

#### 3.1.2. Description of main variables

##### 3.1.2.1. Dependent variable: Risk-averse attitudes

Existing studies on risk attitudes have mainly used questionnaires to construct risk attitude variables based on respondents’ answers to relevant test questions (Charness and Viceisza, 2016). Risk attitudes derived from subjective evaluations of individual risk attitudes have also been shown to have good explanatory power for objective risk behaviors (Guiso and Paiella, 2008). Referring to the research of Xie et al. (2022), this paper uses the questionnaire on investment risk attitudes from the 2019 CHFS, which asks “If you had a sum of money to invest, which investment project would you prefer most?” The statement is measured on a five-point Likert scale (1 = preference for high risk/high return projects, 5 = not willing to take any risk). Generally, the more individuals choose low-risk projects when making investments, the more risk-averse they are. Therefore, we reencoded the data based on a five-point Likert Scale (1 = very low-risk aversion, 5 = very high-risk aversion).

##### 3.1.2.2. Independent variable: Contract performance rate

Contract performance rates is the speed at which a contracting party fulfills their contractual duties. Performance contracts mainly affect enterprises in the industrial chain that deliver products or funds on a schedule according to specific contract requirements. On one hand, the contract performance rates of upstream companies in the supply chain affect those of downstream enterprises (Tang et al., 2007). On the other hand, in practice, the entrepreneurs’ contract execution rates will likewise affect their perception of the overall contract performance rate of the industrial chain. Therefore, this study uses the questions in 2019 CHFS household questionnaire that ask: “How long is the average payback period of your accounts receivable?” and “How long is the average period of your credit sales?” Both two statements are measured on a six-point scale (1 = within 1 month, 6 = more than 2 years). The obtained values are weighted and rescaled to construct the variables that represent the contract performance rate. The converted values are between 1 and 12, and the larger the value, the higher the contract performance rate.

##### 3.1.2.3. Mediating variable: Subjective well-being

Referring to Huang (2022), this paper uses a question measuring subjective well-being from the 2019 CHFS, which asks: “Generally speaking, do you feel happy now?” The statement is measured on a five-point Likert scale (1 = very unhappy, 5 = very happy). Although this measure is relatively brief, the research has shown that it is psychometrically adequate (Veenhoven and Ehrhardt, 1995) and has adequate validity and reliability (Krueger and Schkade, 2008).

##### 3.1.2.4. Moderating variable: Regional business environment

The World Bank defines *Doing Business* as the condition in the external environment faced by businesses throughout their life cycle (The World Bank, 2017). Also, the World Bank has set up a system to evaluate the business environment. However, this evaluation system has certain limitations considering it focuses too much on the government approval process and excludes key factors such as market size and infrastructure. Therefore, this paper refers to the existing research (e.g., Zhang et al., 2020) to incorporate more dimensions into the existing evaluation system and assign weights to secondary indicators through text analysis (Grimmer and Stewart, 2013).

The modified system is shown in Table 1.

**Table 1:** Business environment index system for Chinese provinces.

Target layer	Guideline layer: Tier 1 indicators	Indicator layer: Tier 2 indicators
Regional business environment (100%)	Investment environment (44.16%)	Bank loan-to-deposit ratio (0.20%)
		Non-performing loan ratio (0.20%)
		Government financial regulatory expenditures (8.06%)
		Foreign direct investment (7.77%)
		Number of listed companies (5.49%)
		Trade dependence (5.33%)
		Number of foreign enterprises investment (3.63%)
		Registered capital of foreign-invested enterprises (5.10%)
		Market capitalization (8.38%)
	Market environment (25.81%)	R&D investment (2.26%)
		Number of scientific research institutions (0.57%)
		R&D output (6.87%)
		Percentage of non-state economy (0.15%)
		Number of employees in the non-state economy (1.78%)
		Land price (0.37%)
		Commercial real estate share (0.86%)
		Percentage of advertising agencies (9.33%)
		Number of lawyers per million people (3.62%)
	Government intervention (4.57%)	Crime rate of government personnel in office (0.60%)
		Government revenue (0.29%)
		Government financial self-sufficiency rate (0.89%)
		Administrative fee income (0.94%)
		Central government subsidy income (1.34%)
		Government workers as a percentage (0.51%)
	Population consumption environment (9.13%)	Years of schooling *per capita* (0.07%)
		Average age of the population (0.79%)
		*Per capita* income of residents (0.86%)
		*Per capita* consumption expenditure (0.71%)
		Level of urbanization (0.30%)
		Population density (6.40%)
	Public service environment (16.33%)	Internet penetration rate (0.60%)
		Road density (1.98%)
		Rail density (3.00%)
		Number of government investments in industrial pollution control (8.11%)
		The investment amount of pollution treatment per unit area (2.64%)

The numbers in parentheses represent the weight of the indicator.

Values were calculated to characterize the business environment of each province according to the indicators applied in this research. The raw data used to evaluate the local business environment were obtained from the Almanac of China’s Finance and Banking (2020) and China Statistics Yearbook (2020). The results are shown in Table 2.

**Table 2:** 2019 Business environment evaluation results by province in China.

Province	Index	Province	Index	Province	Index
Anhui	0.0997	Chongqing	0.1635	Sichuan	0.0823
Beijing	0.3729	Jiangxi	0.1016	Tianjin	0.2613
Fujian	0.1277	Liaoning	0.1907	Yunnan	0.0583
Gansu	0.0860	Neimenggu	0.1496	Zhejiang	0.2253
Guangdong	0.2757	Ningxia	0.1153	Heilongjiang	0.0634
Guangxi	0.0823	Qinghai	0.0709	Hubei	0.0958
Guizhou	0.0946	Shandong	0.3241	Hunan	0.1210
Jiangsu	0.1852	Shanxi	0.1131	Jilin	0.1303
Hebei	0.1021	Shaanxi	0.1106	Hainan	0.2332
Henan	0.0988	Shanghai	0.5123		

##### 3.1.2.5. Control variables

Following existing studies (e.g., Hartog et al., 2002; Dohmen et al., 2011), this research classifies the factors that may affect entrepreneurs’ risk attitudes into four categories, including individual, family, firm, and regional characteristics. The individual characteristics include age, age squared, gender, education level, and health status. The family characteristics include household income and expenditures. The firm characteristics are expressed by firm size. Finally, the regional characteristics include a province score, which assigned as 1, 2, 3… 29 by province and used as the regional fixed effect in the econometric regression model.

The descriptive statistics of the above variables are shown in Table 3.

**Table 3:** Descriptive statistics of the main variables.

Variable name	Variable definition	Sample size	Average value	Standard deviation
*Explained variables*				
Risk aversion attitudes	Very low risk aversion = 1	3,660	3.994	1.153
	Very high risk aversion = 5			
*Explanatory variables*				
Contract performance rate	The weighted sum of accounts receivable and accounts payable after convert	3,570	4.058	2.491
*Mediating variables*				
Subjective well-being	Very unhappy = 1; very happy = 5	3,656	2.232	0.870 6
*Moderating variables*				
Regional business environment	2019 Business environment evaluation results by province in China	3,649	0.146	0.079
*Control variables*				
Gender	Male = 1; Female = 0	3,660	0.507	0.500 1
Age	2019-Year of birth	3,660	41.420	12.341
Age squared	Age^*^Age	3,660	1867.88	1021.630
Health status	Very poor = 1; very healthy = 5	3,658	2.317	0.930 1
Education level	Illiterate = 1; doctoral degree = 9	3,650	3.989	1.674
Household income	Total household income in 2018, taking the pairwise treatment	3,431	11.291	1.354
Household expenditure	Monthly household expenditures for 2018, taken as a pair	3,372	5.333	1.095
Firm size	Number of employees, logarithmzed	3,565	0.578	0.991
Province indicator	Value assigned by province (1–29)	3,649	-	−1 27

### 3.2. Methodology

#### 3.2.1. Benchmark model

Based on the previous theoretical analysis, we construct the following estimation model to test the impact of contract performance rates on entrepreneurs’ risk attitudes:


(1) $$Aversion_{i}=\alpha_{0}+\alpha_{1}Rate_{i}+\alpha_{2}X_{i}+Province_{j}+\varepsilon_{i}$$

In Equation (1), *Aversion* is the entrepreneurs’ risk aversion level, *Rate* is the core explanatory variable of the contract performance rate, *X* is a series of control variables that may influence the dependent variable, including the individual characteristics of entrepreneurs and household characteristics, *Province* is the province indicator, which serves as a regional fixed effect, and ε denotes the random error term.

#### 3.2.2. Mediating-effect model

To identify potential mediating effect, this paper constructs Equations (2) and (3) based on Equation (1).


(2) $$\begin{array}{c} SWB_{i}=\beta_{0}+\beta_{1}Rate_{i}+\beta_{2}X_{i}+Province_{j}+v_{i} \end{array}$$

(3) $$\begin{array}{c} Aversion_{i}=\gamma_{0}+\gamma_{1}Rate_{i}+\gamma_{2}SWB_{i}+\gamma_{3}X_{i}+Province_{j}+u_{i} \end{array}$$

Equation (2) responds to the effect of contract performance rates on entrepreneurs’ subjective well-being. *SWB* represents the mediating variable of subjective well-being, and *β*_1_ indicates its effect. Equation (3) represents the effect of contract performance rates as well as the mediating variable of entrepreneurs’ risk aversion, and parameter *γ*_2_ represents the effect of the mediating variable of risk aversion. The description of the other variables in Equations (2) and (3) are consistent with those in Equation (1). According to the existing studies (e.g., Bollen, 2012), the mediating effect can be identified through the following process:

In the first step, *α*_1_ in Equation (1) is tested for significance. If it is significant, the second step of analysis is conducted; otherwise, the analysis is stopped.

According to related studies (e.g., Baron and Kenny, 1986; Batrancea and Nichita, 2015), in the second step, this paper first tests whether *β*_1_ in Equation (2) is significant. Second, *γ*_2_ in Equation (3) is tested for significance. If both coefficients are significant, the third step of the test is conducted. The fourth step is conducted if at least one of the above two coefficients is statistically nonsignificant.

Referring to a related study (Judd and Kenny, 1981), in the third step, this paper first checks whether *γ*_1_ in Equation (3) is no longer significant; if so, the mediating effect of subjective well-being on contract performance rates and entrepreneurs’ risk aversion level has a full mediating effect. Otherwise, the mediating effect of subjective well-being on contract performance rates and entrepreneurs’ risk aversion level has a partial mediating effect.

Referring to a related study (Sobel, 1982), this paper further goes on to identify the potential mediating effect using the Sobel test in the fourth step. Passing the Sobel test indicates that the mediating effect exists. If it fails to pass the test, there is no mediating effect. The Sobel test formula is as follows:


(4) $$\begin{array}{c} Z=\frac{\beta_{1}\gamma_{2}}{\sqrt{\beta_{1}^{2}S_{\gamma }^{2}+\gamma_{2}^{2}S_{\beta }^{2}}} \end{array}$$

where *S_γ_* and *S_β_* are the standard deviations of the estimated values of parameters *γ*_2_ and *β*_1_, respectively.

#### 3.2.3. Moderating and mixed-effect models

The research hypotheses argue that the impact of contract performance rates on entrepreneurs’ risk attitudes may be moderated by the regional business environment. To identify this potential moderating effect, we estimate the following regression model:


(5) $$\begin{array}{c} \begin{array}{l} \;Aversion_{i}=\gamma_{4}+\gamma_{5}Rate_{i}+\gamma_{6}Business_{i}+\gamma_{7}\\ Rate_{i}\times Business_{i}+\gamma_{8}X_{i}+Province_{j}+u_{i} \end{array} \end{array}$$

where *Business* represents the potential moderating variables measured as the regional business environment and *γ*_6_ indicates its effect. The other variables in the model are the same as in the previous equations. According to the existing studies (e.g., Batrancea et al., 2018; Jian et al., 2021), whether the regulatory effect exists can be identified by the significance of the coefficient on the interaction term. If it is significant, the regulatory effect exists; otherwise, it does not. Hence, this paper identifies the moderating effect of the regional business environment on contract performance rates and entrepreneurs’ risk attitudes through the following process:

In the first step, this paper constructs an interaction term using the product of the variables *Rate* and *Business* and includes it in the regression model, as shown in Equation (5). In the second step, this paper tests whether *γ*_7_ in Equation (5) is significant. If it does, the moderating effect exists; otherwise, it is absent. At the same time, if *γ*_7_ is less than 0, it has a negative effect.

To further verify the above mediating and moderating effects, this research constructs and estimates the following mixed-effect model:


(6) $$\begin{array}{c} \begin{array}{l} \;Aversion_{i}=\\ \rho_{1}+\rho_{2}Rate_{i}+\rho_{3}SWB_{i}+\rho_{4}Business_{i}+ \end{array}\\ \rho_{5}Rate_{i}\times Business_{i}+\rho_{6}X_{i}+Province_{j}+\pi_{i}. \end{array}$$

The dependent variable in this paper is entrepreneurs’ risk-averse attitudes (risk aversion level) constructed from the question concerning investment propensity in the CHFS questionnaire. It varies according to five-level ordered category variables. This research also refers to the practice of most studies (e.g., Hassen, 2018; Yu et al., 2020) and selects the ordered probit (oprobit) model for regression estimation. However, some scholars have employed the ordinary least squares (OLS) and ordered logit (ologit) models for systematic categorical variable analysis (Ghader et al., 2019; Zhang et al., 2021a). Therefore, the regression results of the OLS and ologit models were used as a robustness check to verify the reliability of the results in this paper.

## 4. Empirical results

### 4.1. Direct effects analysis from ordered probit

Table 4 reports the estimated results of the benchmark Equation (1), which measures the impact of contract performance rates on entrepreneurs’ risk attitudes. Equations (1)–(1) introduces the core independent variable of the effect of contract performance rates into the benchmark model. Equations (1)–(2) includes the respondents’ individual variables based on Equations (1)–(1). Equations (1)–(3) further includes the control variables of respondents’ family characteristics based on Equations (1)–(2). Equations (1)–(4) adds the enterprise variables to Equations (1)–(3). Equations (1)–(5) also accommodates the regional fixed effect variable based on Equations (1)–(4). The regression coefficients on the contract performance rate variables in all five models are positive. This implies that the null hypothesis can be rejected at the 1% significance level, thus indicating that the positive response to contract performance rates can significantly strengthen entrepreneurs’ risk-averse attitudes and supporting Hypothesis 1.

**Table 4:** Regression results of the direct effect analysis from the ordered probit model.

	(1–1)	(1–2)	(1–3)	(1–4)	(1–5)
Contract performance rate	0.025^***^	0.030^***^	0.030^***^	0.026^***^	0.026^***^
	(0.0074)	(0.0074)	(0.0080)	(0.0080)	(0.0080)
Gender		0.025	0.013	0.012	0.010^***^
		(0.0374)	(0.0398)	(0.0400)	(0.0400)
Age		−0.055^***^	−0.037^***^	−0.034^***^	−0.033^***^
		(0.0102)	(0.0109)	(0.0110)	(0.0110)
Age squared		0.000^***^	0.000^***^	0.000^***^	0.000^***^
		(0.0001)	(0.0001)	(0.0001)	(0.0001)
Health status		0.002	−0.030	−0.040	−0.428
		(0.0216)	(0.0234)	(0.0235)	(0.0235)
Education level		−0.106^***^	−0.072^***^	−0.057^***^	−0.057^***^
		(0.0128)	(0.0140)	(0.0143)	(0.0143)
Household income			−0.081^***^	−0.056^***^	−0.056^***^
			(0.0160)	(0.0165)	(0.0165)
Household expenditure			−0.116^***^ (0.0191)	−0.102^***^	−0.102^***^
				(0.0193)	(0.0193)
Firm size				−0.122^***^	−0.123^***^
				(0.0222)	(0.0222)
Fixed effect					YES
Sample size	3,570	3,559	3,128	3,113	3,107
Pseudo *R*^2^	0.0012	0.0155	0.0229	0.0264	0.0266

Robust standard errors are reported in parentheses. ^***^p < 0.01, ^**^p < 0.05, ^*^p < 0.1.

The results in terms of the control variables are as follows: gender, age, age squared, education level activities, household income, household expenditure, and firm size have an impact entrepreneurs’ risk-averse attitudes, which is consistent with other research conclusions (e.g., Gollier, 2002; Guiso and Paiella, 2008; Kapteyn and Teppa, 2011; Hetschko and Preuss, 2020). However, the other control variables were not significant.

### 4.2. Mediating-effect analysis

Table 5 reports the estimation results of Equations (1)–(3), which measure the mediating effect of contract performance rates on entrepreneurs’ risk aversion. Equation (2) reports the impact of the core explanatory variable (contract performance rate) on the mediating variable (subjective well-being). Equation (3) reports the regression results of after adding and estimating the explanatory and mediating variables simultaneously. From the estimation results, it can be seen that the regression coefficients on the core explanatory variable of contract performance rates on subjective well-being are positive and statistically significant at the 1% level, thus indicating that improving contract performance rates can significantly improve entrepreneurs’ subjective well-being. After adding and estimating the explanatory and mediating variables simultaneously, the regression coefficients reject the null hypothesis at the 1% significance level. According to the identification criteria, the mediating effect exists, whereby improving contract performance rates can reinforce entrepreneurs’ risk aversion by improving their subjective well-being. Hypothesis 2 is therefore verified.

**Table 5:** Regression results of the mediating effect using the oprobit model.

	(1)	(2)	(3)
Contract performance rate	0.026^***^	0.023^***^	0.024^***^
	(0.0079)	(0.0078)	(0.0081)
Subjective well-being			0.090^***^
			(0.0238)
Individual characteristics	YES	YES	YES
Family characteristics	YES	YES	YES
Enterprise characteristics	YES	YES	YES
Fixed effect	YES	YES	YES
Sample size	3,107	3,107	3,103
Pseudo *R*^2^	0.0266	0.0191	0.0282

Robust standard errors are reported in parentheses. ^***^p < 0.01, ^**^p < 0.05, ^*^p < 0.1.

### 4.3. Moderating and mixed-effect analysis

Table 6 reports the estimated results of Equations (5) and (6). Equations (5–1) and (5–2) are the estimation results after adding the core explanatory and potential moderating variables simultaneously. Equation (6) is a mixed-effects model with mediating and moderating variables. According to the moderating effect test method, the interaction of the term coefficient in Equations (5–2) is negative, and the null hypothesis is rejected at the 5% significance level, thereby registering the existence of a moderating effect. The regional business environment factor plays a negative moderating role in the relationship between contract performance rates and entrepreneurs’ risk aversion. Thus, Hypothesis 3 is supported.

**Table 6:** Oprobit regression results of the moderating and mixed effects.

	Moderation effect			Mixed effect
	(1)	(5–1)	(5–2)	(6)
Contract performance rate	0.026^***^	0.025^***^	0.058^***^	0.057^***^
	(0.0079)	(0.0081)	(0.0174)	(0.0174)
Subjective well-being				0.092^***^
				(0.0238)
Regional business environment		0.611	−0.281	−0.267
		(0.2697)	(0.4910)	(0.4910)
Contract performance rates × Regional business environment			−0.231^**^	−0.234^**^
			(0.1066)	(0.1067)
Individual characteristics	Yes	Yes	Yes	Yes
Family characteristics	Yes	Yes	Yes	Yes
Enterprise characteristics	Yes	Yes	Yes	Yes
Fixed effect	Yes	Yes	Yes	Yes
Sample size	3,107	3,107	3,107	3,103
Pseudo *R*^2^	0.0266	0.0272	0.0278	0.0295

Robust standard errors are reported in parentheses. ^***^p < 0.01, ^**^p < 0.05, ^*^p < 0.1.

## 5. Robustness check

The above empirical analyses substantially address the research questions and hypotheses developed in this paper. However, to ensure the reliability and stability of the results, the ensuing section further tests the robustness of the empirical results by applying different estimation techniques. Therefore, the OLS and ologit models have been implemented to verify the reliability of the results.

### 5.1. Core explanatory variables for robustness check

The core explanatory variable of this research, contract performance rates, is derived from the weighted sum of the accounts receivable and the average collection time of the accounts payable of the entrepreneur’s enterprise. Moreover, whether the entrepreneur’s business experiences overdue accounts receivable can also reflect the contract performance rates in the supply chain to a certain extent (Wang et al., 2023). Therefore, in this section, the core explanatory variable is replaced with “Has your company experienced overdue accounts receivable?” and then estimated based on Equation (1). The original data are obtained from the question “Has your company experienced overdue accounts receivable?” in the 2019 China Household Finance Survey household questionnaire (1 = Yes, 0 = No). The obtained results are shown in Column (1) of Table 7. Comparing the current results with those in Table 4 shows that the significance levels as well as the positive and negative signs of the coefficients do not change after replacing the explanatory variables. This indicates that the estimating models employed earlier are robust and reliable.

**Table 7:** Robustness test results.

	(1)	(2)	(3)	(4)	(5)	(6)
	Risk-averse attitudes	Subjective well-being	Risk-averse attitudes	Risk-averse attitudes	Risk-averse attitudes	Risk-averse attitudes
Past due accounts receivable	0.249^***^					
	(0.0557)					
Contract performance rate		0.037^***^	0.037^***^	0.035^***^	0.086^***^	0.083^***^
		(0.0134)	(0.0135)	(0.0136)	(0.0296)	(0.0297)
Subjective well-being				0.149^***^		0.152^***^
				(0.402)		(0.0403)
Regional business environment					−0.408	−0.364
					(0.8337)	(0.8338)
Interaction effect					−0.352^*^	−0.350^*^
					(0.1820)	(0.1822)
Individual characteristics	YES	YES	YES	YES	YES	YES
Family characteristics	YES	YES	YES	YES	YES	YES
Enterprise characteristics	YES	YES	YES	YES	YES	YES
Fixed effect	YES	YES	YES	YES	YES	YES
Sample size	2,680	3,103	3,107	3,103	3,107	3,103
Pseudo *R*^2^	0.0299	0.0197	0.0291	0.0306	0.0301	0.0316

Robust standard errors are reported in parentheses. ^***^p < 0.01, ^**^p < 0.05, ^*^p < 0.1.

### 5.2. Estimation methods for robustness check

This research also used the ologit estimation methods to conduct regression analyses on the empirical model as shown in Columns (2)–(6) of Table 7. From the regression results, it can be seen that the explanatory variables, regression coefficients, and significance of the mediating and moderating variables did not change significantly. This implies that key results of this study—the direct, mediating, and moderating effects—are consistent and robust.

## 6. Heterogeneity analysis

China, as an emerging economy, has faced challenges in closing the gap between urban and rural communities in terms of socioeconomic and infrastructural development. Hence, in this study we test how regional differences influence the effect of contract performance rates on entrepreneurs’ risk-averse attitudes. Moreover, there is some heterogeneity within the group of entrepreneurs. Several studies have shown that prior work experience affects individual risk attitudes. To address these problems, we grouped the sample according to whether the entrepreneur lived in the eastern region, in a rural area, or was a first-time entrepreneur and ran separate regressions for each group. The results are shown in Table 8.

**Table 8:** Heterogeneity analysis results.

	Region		Entrepreneurial experience		Urban and rural	
	(1)	(2)	(3)	(4)	(5)	(6)
	Eastern region	Non-Eastern Region	First time	Not the first time	Urban	Rural
Contract performance rate	0.026^***^	0.024^**^	0.021^**^	0.024^*^	0.017	0.031^***^
	(0.0125)	(0 0.0106)	(0.0100)	(0.0138)	(0.0135)	(0 0.0101)
Individual characteristics	YES	YES	YES	YES	YES	YES
Family characteristics	YES	YES	YES	YES	YES	YES
Enterprise characteristics	YES	YES	YES	YES	YES	YES
Fixed effect	YES	YES	YES	YES	YES	YES
Sample size	1,299	1808	2,201	906	1,102	1,998
Pseudo *R*^2^	0.0345	0.0312	0.0278	0.0194	0.0320	0.0271

Robust standard errors are reported in parentheses. ^***^p < 0.01, ^**^p < 0.05, ^*^p < 0.1.

As shown in Columns (1)–(4) of Table 8, the effect of contract performance rates on entrepreneurs’ risk-averse attitudes did not change significantly in response to changes in location or the level of entrepreneurial experience. However, Columns (5) and (6) of Table 8 report an exciting result. This research finds that the regression coefficient on contract performance rates for rural entrepreneurs is significantly positive, while that for urban entrepreneurs is statistically nonsignificant. This implies that an increase in contract performance rates only has a significantly positive effect on the risk aversion level of rural entrepreneurs. In contrast, the risk attitudes of urban entrepreneurs are not related to contract performance rates. Further combining the regression results of the regional and entrepreneurial experience heterogeneity tests, it can be inferred that contract performance rates have a significantly positive effect on entrepreneurs’ risk-averse attitudes, which is mainly driven by rural entrepreneurs. The possible reasons for this are threefold.

First, the difference in the regional business environment between urban and rural areas may account for this finding. The imbalance in China’s economic development is reflected more in urban and rural areas than in eastern and non-eastern regions. As of now, there is still a significant gap in the overall level of infrastructural development and public services between urban and rural areas in China (Li and Liu, 2021). As a result, entrepreneurs in rural areas have less access to resources and market information than urban entrepreneurs. Combined with the regression results of Equations (5–2) shown in Table 6 in the previous section, the business environment plays a negative moderating role in the effect of contract performance rates on entrepreneurs’ risk attitudes. In turn, the business environment tends to be worse in rural areas. Hence, the results show a more significantly positive effect of contract performance rates on the rural entrepreneurs’ risk attitudes.

Second, employment opportunities are more available in urban areas than in rural areas, so urban entrepreneurs usually engage in entrepreneurial activities as individuals (Chen et al., 2022). Family members of urban entrepreneurs or even the entrepreneurs themselves may continue to work for pay while operating their own business. In contrast, rural entrepreneurs are mostly family based because of the relatively few employment opportunities in rural areas. This makes income from entrepreneurial projects the only source of income for most rural entrepreneurial households. Thus, business failure is often unacceptable to rural entrepreneurs. As a result, rural entrepreneurs show lower risk tolerance, and their risk attitudes are therefore more sensitive to the effects of contract performance rates.

Third, compared to urban areas, rural areas in China are more meaningfully influenced by traditional Confucianism. Confucianism is one of the most far-reaching informal systems influencing Chinese society (Chen et al., 2021). In ancient China, Confucianism was used to guide social governance, and the feudal government required everyone to play their role by constructing strict class and identity differences. This strict system of rituals and laws profoundly influenced Chinese society’s cultural and psychological structure, thus making the familiar people comfortable with the status quo and more conservative. Some studies have shown that the greater the exposure to Confucian culture, the lower the level of risk-taking will be (e.g., Jin et al., 2017). Furthermore, this correlation is weaker in regions with high marketization and openness to the outside world. In contemporary society, Chinese society in urban areas is more strongly impacted by foreign culture, while in rural areas, more traditional culture is retained. Thus, cultural differences may also be one of the reasons for rural entrepreneurs’ risk attitudes being more susceptible to contract performance rates.

## 7. Conclusions and policy recommendations

It is essential to study the influencing mechanisms of entrepreneurs’ risk attitudes to gain insight into the investment decisions made by their enterprises. Using the 2019 China Household Finance Survey (CHFS) data matched with the regional business environment index, this paper conducted an empirical study on the impact of contract performance rates on entrepreneurs’ risk attitudes and their influencing mechanisms. From the regression results, this paper concludes that: (1) contract performance rates can significantly strengthen entrepreneurs’ risk-averse attitudes; (2) contract performance rates can enhance entrepreneurs’ subjective well-being and thus indirectly strengthen entrepreneurs’ risk-averse attitudes; (3) the business environment negatively moderates the influence of contract performance rates on the entrepreneurs’ risk-averse attitudes; and (4) increasing contract performance rates significantly strengthens the risk-averse attitudes of rural entrepreneurs, but this effect on urban entrepreneurs appears statistically nonsignificant.

The findings from this study have strong policy implications. Based on the findings, we recommend that relevant policies in China be developed to enhance social and economic activity, optimize regional business environments by taking measures specific to each, and reduce entrepreneurs’ risk aversion. Moreover, the government should pay attention to training and education programs for different types of entrepreneurs and guide entrepreneurs to adequately understand market risks. Finally, efforts should be made to change entrepreneurs’ old-world ideologies to reverse the risk aversion widespread among rural entrepreneurs.

## 8. Limitations and future research prospects

This study has two main limitations that need to be further explored and improved in the future. (1) Cross-sectional data were used in our empirical analyses. In future studies, the impact of time should be considered. Dynamic data on entrepreneurs’ risk attitudes, contract performance rates, and changes in the regional business environment can be obtained through cross-stage and multipoint tracking for more in-depth studies. (2) Due to the limitations in the original data, this study did not develop a further analysis of entrepreneurial heterogeneity. The effect of contract performance rates on risk attitudes may vary across different types of entrepreneurs (e.g., the industry in which they are located, whether they are owners of a family business, and whether they are owners of a publicly traded business). Future research can further develop a more in-depth study of different entrepreneur groups.

## Data availability statement

The datasets presented in this study can be found in online repositories. The names of the repository/repositories and accession number(s) can be found at: https://chfs.swufe.edu.cn/.

## Author contributions

ZS: conceptualization and writing original draft preparation. SL: methodology. MH: data curation and data collection. AV: writing reviewing and editing. CO: software. JZ: funding acquisition and supervision. All authors contributed to the article and approved the submitted version.

## Funding

This research was funded by the National Social Science Foundation of China (19BGL149).

## Conflict of interest

The authors declare that the research was conducted in the absence of any commercial or financial relationships that could be construed as a potential conflict of interest.

## Publisher’s note

All claims expressed in this article are solely those of the authors and do not necessarily represent those of their affiliated organizations, or those of the publisher, the editors and the reviewers. Any product that may be evaluated in this article, or claim that may be made by its manufacturer, is not guaranteed or endorsed by the publisher.
